# Waveguide Characterization of S-Band Microwave Mantle Cloaks for Dielectric and Conducting Objects

**DOI:** 10.1038/srep19716

**Published:** 2016-01-25

**Authors:** Antonino Vitiello, Massimo Moccia, Gian Paolo Papari, Giuliana D’Alterio, Roberto Vitiello, Vincenzo Galdi, Antonello Andreone

**Affiliations:** 1 CNR-SPIN and Department of Physics, University of Naples “Federico II”, I-80125 Naples, Italy; 2 Waves Group, Department of Engineering, University of Sannio, I-82100 Benevento, Italy; 3 MBDA Italia s.p.a., I-80070 Bacoli (NA), Italy

## Abstract

We present the experimental characterization of *mantle cloaks* designed so as to minimize the electromagnetic scattering of moderately-sized dielectric and conducting cylinders at S-band microwave frequencies. Our experimental setup is based on a parallel-plate waveguide system, which emulates a two-dimensional plane-wave scattering scenario, and allows the collection of near-field maps as well as more quantitative assessments in terms of global scattering observables (e.g., total scattering width). Our results, in fairly good agreement with full-wave numerical simulations, provide a further illustration of the mantle- cloak mechanism, including its frequency-sensitivity, and confirm its effectiveness both in restoring the near-field impinging wavefront around the scatterer, and in significantly reducing the overall scattering.

The quest for “invisibility” is a longstanding research topic which has always fascinated researches in various fields of physics (see, e.g., ref. 1 for a recent review). In a broad sense, the problem can be posed as the *suppression* of the scattering signature due to an interrogating wave signal impinging on a given object.

In electromagnetic (EM) scenarios, of specific interest for this study, concepts such as “neutral” inclusions as well as “invisible” sources, scatterers, and antennas have been investigated since the 1960s (see, e.g., refs 2, 3, 4, 5, 6, 7, 8), but a major revamp has taken place during the last decade, associated with the suggestive term of “cloaking” and catalyzed by the advances in the field of artificial materials and “metamaterials.” Within this framework, prominent approaches are based on the *scattering cancellation* concept^9^ (and its possible plasmonic–10,11,12 and mantle-based^13^ implementations) and the *transformation-optics* paradigm^14^,^15^,^16^ (and its possible non-Euclidean^17^ and carpet-type^18^,^19^,^20^ variants). Alternative approaches also worth of mention are those based on anomalous localized resonances^21^, transmission-line networks^22^, parallel-plate structures^23^, and topology optimization^24^,^25^,^26^. Also, strategies based on ray optics (for incoherent natural light)^27^,^28^, active sources^29^,^30^, non-Foster elements^31^, and parity-time-symmetric configurations^32^,^33^ have been explored in order to overcome certain inherent limitations of passive, material-based schemes (see, e.g., ref. 34) and/or to achieve otherwise unattainable effects (e.g., unidirectionality).

More recently, a growing interest has been elicited by applications to dc (electrostatic and magnetostatic) scenarios^35^,^36^,^37^,^38^ and diffuse light^39^, as well as to other physical domains including acoustics^40^,^41^,^42^,^43^, elastodynamics^21^,^44^,^45^, liquid surface waves^46^, quantum matter waves^47^, and thermodynamics^48^,^49^,^50^. Moreover, applications to *multiphysics* scenarios (e.g., thermal/electric^51^,^52^, also in conjunction with other functionalities^53^,^54^) seem also very promising.

Overall, cloaking has rapidly become a fast-pacing multidisciplinary research topic, with a wealth of potentially disruptive practical applications, besides the more or less obvious invisibility and camouflaging, ranging from the reduction of antenna interference^55^,^56^ to noninvasive sensing^57^,^58^ and thermal management^48^,^49^,^50^. The reader is referred to refs 59,60 for recent comprehensive reviews.

In this paper, we focus on the *mantle-cloak* approach. Originally proposed in ref. 13 as a possible implementation of the scattering-cancellation strategy, and experimentally validated at microwave frequencies^61^,^62^,^63^, this approach conceptually relies on the design^64^,^65^ of a reactive *metasurface* whose response, at a given design frequency, can cancel out the lowest-order multipolar contribution to the scattering signature. By comparison with the *plasmonic* implementation^10^,^11^,^12^, mantle cloaks tend to be particularly suited for microwave and terahertz frequencies, providing low-profile, conformal, easy-to-fabricate configurations that are especially attractive for applications to reduction of antenna coupling^56^,^66^,^67^. Moreover, tunability mechanisms can be implemented using varactor-diode-loading^68^ or advanced materials like graphene (in the form of monolayers^69^ or nanostructured metasurfaces^70^,^71^,^72^). Very recently, *graded* metasurfaces have also been applied to carpet-cloaking and wavefront-restoration^73^,^74^.

Against this background, in our study, we complement the experimental results in refs 61, 62, 63 by presenting the characterization of mantle-cloak prototypes for dielectric and conducting cylindrical objects operating at S-band (3 GHz) microwave frequencies. More specifically, by means of a custom parallel-plate-waveguide scanner, we measure (and compare) the near-field maps in the absence and presence of the cloaked and uncloaked (bare) objects, as well as the total scattering width (SW).

Results clearly show the cloak-induced restoration of the near-field wavefronts and the consequent reduction in the total SW of nearly 99% (20 dB) and 80% (7 dB) for the dielectric and conducting case, respectively, in fairly good agreement with full-wave numerical simulations.

## Results

**Figure 1:**
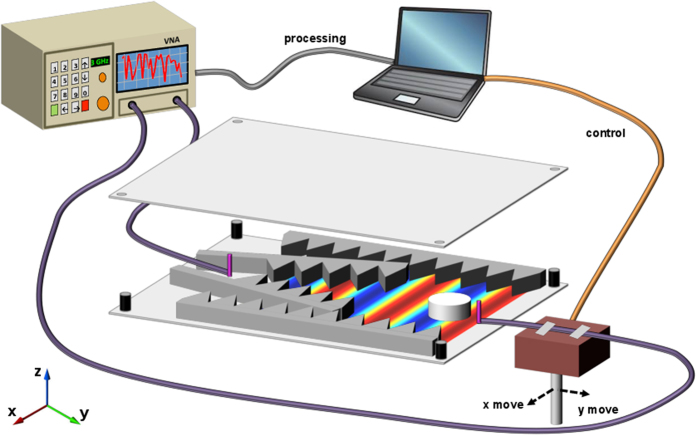
Three-dimensional cutaway sketch of the S-band parallel-plate measurement setup utilized (see the Methods section for details).

Similar to other experimental demonstrations of EM cloaking^63^, lensing^75^ and transmission^76^ through artificial materials in the microwave region, our measurements are performed in a parallel-plate waveguide setup (see Fig. 1 for a schematic). Such configuration allows for the emulation of an environment with an infinite extent along one direction (*z*-axis, in our case). Over the S-band frequency range of interest, the waveguide supports a single transverse-EM (TEM) mode with *z*-polarized (i.e., normal to the plates) electric field. The restricted polarization in two dimensions makes EM measurements somewhat easier, since cross-polarization can be neglected and the scattering problem becomes scalar in character. Small monopole antennas are used to excite the waveguide and sample the field distribution. To this aim, the probe antenna is placed on a translation stage that allows to scan an area of 70 mm × 50 mm (or, in terms of wavelength, 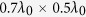 at the nominal frequency 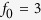 GHz) in the 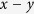 plane. Measurements are recorded using a Vector Network Analyzer (VNA) with port 1 connected to the source antenna and port 2 to the probe. In each measurement, the magnitude and phase of the transmission coefficient 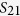 from port 1 to port 2 at each position are recorded, yielding a relative map of the complex electric field as a function of space. Details of the measurement setup are thoroughly described in the Methods section below.

**Figure 2:**
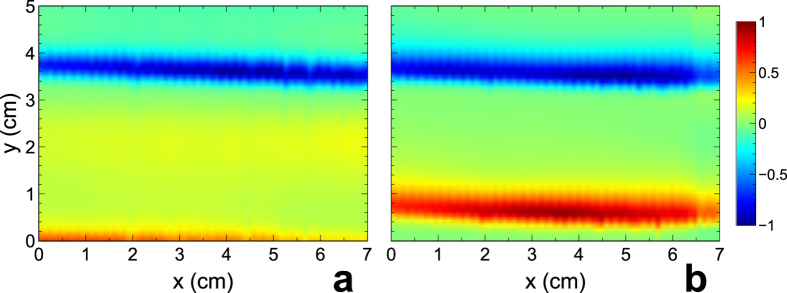
(**a**,**b**) Measured spatial distributions of electric field 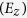 real-part in the empty waveguide (i.e., in the absence of scatterers) at the nominal design frequency 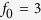 GHz and at 4 GHz, respectively. The waveguide is excited by a monopole antenna located at 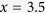 cm, *y* = −57.5 cm (see the Methods section for more details), and thus the quasi-planar wavefronts propagate along the positive *y*-direction. The field values are suitably normalized for better visibility, and displayed in false-color scales.

First, we measured the field map of the empty waveguide far from the source at 3 GHz and 4 GHz, in the region where we intend to place the scatterers. Results are shown in Fig. 2 in terms of the real-part of the electric field 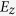, which is directly derived from the above magnitude and phase measurements. Results basically show that: *i)* at reasonable distance from the source 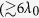 and in the cases shown in the figure), the wavefront is approximately planar; and *ii)* the presence of the (subwavelength) probe in the scanned area does not significantly perturb the field distribution.

**Figure 3:**
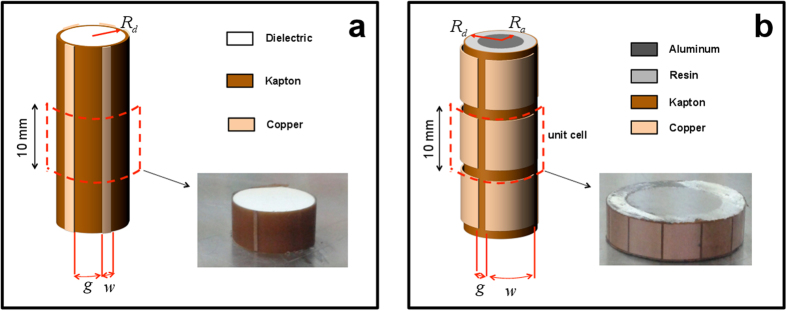
(**a**) Geometry (not in scale) of a dielectric cylinder of radius 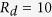 mm covered by a metasurface made of metallic (copper) strips of width 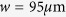 and thickness 17 *μ*m, with gaps 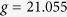 mm, laid on a 100 *μ*m-thick adhesive (Kapton) substrate. Also shown is a photo of the fabricated prototype of finite (10 mm) thickness. (**b**) Geometry (not in scale) of a conducting (aluminum) cylinder of radius 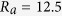 mm covered by a metasurface made of metallic (copper) conformal square patches of sidelength 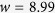 mm with gaps 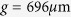 laid on a 100 *μ*m-thick adhesive (Kapton) substrate. In this case, an additional dielectric (resin) spacer of thickness 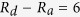 mm separates the cylinder from the metasurface. Also shown is a photo of the fabricated prototype of finite thickness (10 mm, i.e., one unit cell along the *z*-direction). Details on the design and fabrication parameters, as well as the constitutive parameters utilized in the numerical simulations, are provided in the Methods section.

The scatterers under investigation are a dielectric and a conducting homogeneous cylinders, with moderate electrical sizes (diameters 0.2*λ*_0_ and 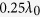, respectively) and same height as the waveguide. They are wrapped with metasurfaces made of thin metallic strips or patches laid on thin adhesive substrates, which act as mantle cloaks at 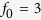 GHz. In the conducting-cylinder case, an additional dielectric spacer is utilized. Figure 3 shows the sketches (not in scale) of the two structures and photos of the fabricated prototypes, with the relevant parameters given in the caption. Details on the mantle-cloak design and prototype fabrication are provided in the Methods section below.

**Figure 4:**
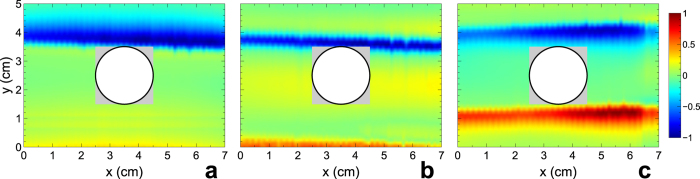
Measured (real-part) electric-field maps pertaining to the dielectric cylinder (cf.Fig. 3a). (**a**) Bare (i.e., uncloaked) cylinder at the nominal design frequency 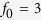 GHz. (**b**,**c**) Cloaked cylinder at 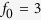 GHz and at a frequency (4 GHz) outside the cloaking band, respectively. The illumination impinges along the positive *y*-direction. The field values are suitably normalized for better visibility, and displayed in false-color scale. The grey-shaded squares delimit regions unaccessible to the probe antenna in view of their close proximity to the scatterers (delimited by thick black circular contours).

Figure 4a shows a field map in the presence of the *bare* (i.e., uncloaked) dielectric cylinder at large distance from the illuminating monopole source. As expected, the presence of the scatterer in the waveguide perturbs the wavefront, introducing a visible additional scattering, especially in the forward direction. The grey-shaded region in the plot delimits the area not scanned by the probe antenna in view of its close proximity to the scatterer. Figure 4b shows the same field map, but in the presence of a *cloaked* dielectric cylinder, at the nominal design frequency 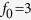 GHz. The observed wavefront is much better defined and more uniform than the previous (uncloaked) case. By comparison with the empty-waveguide results (cf. Fig. 2a), we note that the presence of the mantle cloak drastically reduces the scattering from the cylinder, effectively restoring the almost-planar wavefront. The frequency sensitivity of the cloaking mechanism is confirmed by the field map shown in Fig. 4c, pertaining to a frequency (4 GHz) far away from the nominal design frequency. In this case, the presence of the metasurface is rather ineffective for the cloaking mechanism, and a visible distortion is introduced in the impinging wavefront (compare with Fig. 2b).

**Figure 5:**
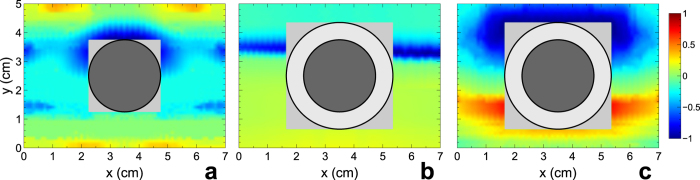
Measured (real-part) electric-field maps pertaining to the conducting cylinder (cf. Fig. 3b). (**a**) Bare (i.e., uncloaked) cylinder at the nominal design frequency 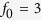 GHz. (**b**,**c**) Cloaked cylinder at 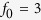 GHz and at a frequency (4 GHz) outside the cloaking band, respectively. The illumination impinges along the positive *y*-direction. The field values are suitably normalized for better visibility, and displayed in false-color scale. The grey-shaded squares delimit regions unaccessible to the probe antenna in view of their close proximity to the scatterers (delimited by thick black circular contours); the different size in panels (**b**,**c**) is due to the presence of an additional 6 mm-thick dielectric spacing layer (cf. Fig. 3b).

In Fig. 5, the same results are shown for the conducting cylinder. Once again, the bare cylinder inside the waveguide scatters the impinging wave in all directions, producing a significant distortion in wavefront (Fig. 5a). Differently from the previous (dielectric) case (cf. Fig. 4a), now the distinctive shadow region, as well as the scattering in the other directions, created by the cylinder is clearly seen in the field map. In the presence of the cloak, at the nominal design frequency, the scattering is drastically suppressed all around the object, even in the forward direction (Fig. 5b). In other words, the mantle cloak allows restoring the impinging wavefront even in the “shadow” region behind the scatterer. The resulting wavefront is hardly distinguishable from the one measured in the empty waveguide (cf. Fig. 2a), and it is impressive to observe the restored near-field distribution just outside the thin cloaking layer, further illustrating the scattering cancellation induced by the optimized metasurface. Also in this case, away from the nominal design frequency, the mantle cloak becomes completely ineffective, and a significant scattering is observed (Fig. 5c).

For a more *quantitative* assessment of the qualitative scenarios above, a meaningful observable is the *total* SW^11^,


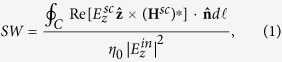


where 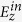 and 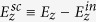 denote the incident and scattered electric field, respectively, 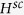 is the scattered magnetic (vector) field, *C* denotes an arbitrary closed contour (with outward unit normal vector 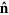, and differential length 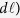 surrounding the scatterer, 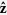 is the *z*-directed unit vector, 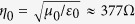 is the vacuum characteristic impedance, and Re and ^*^ denote real-part and complex-conjugation, respectively.

To perform SW measurements, paralleling ref. 11, we measure the incident electric field 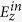 in the empty waveguide, and the total field 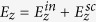 in the presence of the scatterers. From these, we obtain the scattered electric field 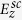 by subtraction, and the corresponding magnetic field 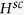 via the relevant Maxwell’s curl equation (by approximating the involved spatial derivatives in terms of finite differences). In particular, our chosen closed contour *C* is the 70 mm × 50 mm rectangle delimiting the field maps in Figs 2,4 and 5, sampled with a 1 mm step. This yields about 240 spatial samples for the numerical quadrature of the contour integral in Eq. (1).

**Figure 6:**
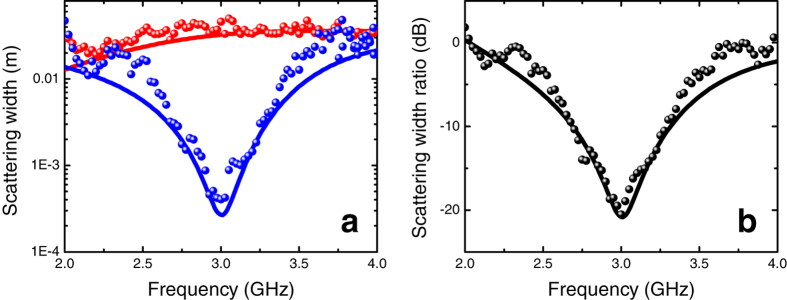
(**a**) SW [cf. Eq. (1)] in semilog scale as a function of frequency for the dielectric cylinder (cf. Fig. 3a) in the absence (red markers) and presence (blue markers) of the mantle cloak. (**b**) Corresponding SW ratio [cf. (2)] in dB scale (black markers). As references, also shown (red, blue and black, solid curves, respectively) are the corresponding predictions from full-wave numerical simulations (see the Methods section for details).

Figure 6a compares the SWs for the bare and cloaked dielectric cylinders, as a function of frequency. For better visualization, a semi-log scale is utilized, and only 80 frequency samples are displayed. Also shown, as references, are the corresponding quantities calculated via full-wave numerical simulations (see the Methods section below for details). As it can be observed, the total SW in the cloaked case is consistently smaller than the uncloaked counterpart within most of the frequency range, with a pronounced dip at the nominal design frequency 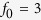 GHz, in fairly good agreement with the numerical predictions. For a more direct assessment, Fig. 6b shows the SW ratio


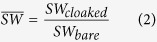


on a dB scale. At the nominal design frequency, the mantle cloak induces a ~20 dB (99%) reduction in the SW. Moreover, a reasonable reduction 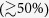 occurs within a 1 GHz frequency band around 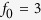 GHz, thereby yielding a virtual “3 dB cloaking bandwidth” of about 33%.

**Figure 7:**
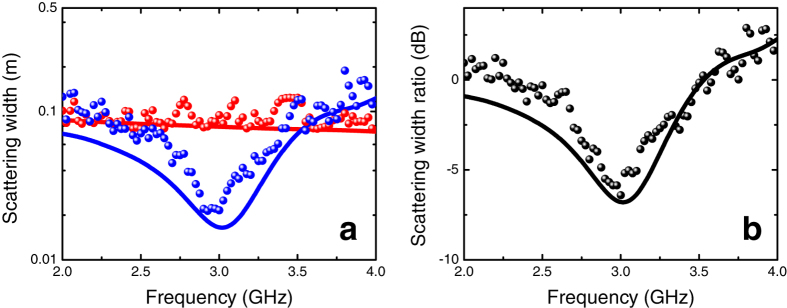
(**a**) SW [cf. Eq. (1)] in semilog scale as a function of frequency for the conducting cylinder (cf. Fig. 3b) in the absence (red markers) and presence (blue markers) of the mantle cloak. (**b**) Corresponding SW ratio [cf. (2)] in dB scale (black markers). As references, also shown (red, blue and black, solid curves, respectively) are the corresponding predictions from full-wave numerical simulations (see the Methods section for details).

The corresponding results for the conducting cylinder are shown in Fig. 7. In this case, the SW reduction observed at the nominal design frequency is nearly 7 dB (80%), with a pronounced asymmetry at higher frequencies, and a mildly narrower “3 dB bandwidth” (500 MHz). Also in this case, a good agreement with the numerical predictions is observed.

## Discussion

The above results confirm that a strong scattering reduction is possible, and is experimentally observed with good accuracy, close to the nominal design frequency 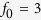 GHz. The application of the mantle cloak on the surface of the scatterers almost fully restores the field distribution observed in the empty waveguide (i.e., in the absence of scatterers) for the case of the dielectric cylinder, and to a lesser extent for the case of the conducting cylinder.

The observed deviations between the measurements and numerical predictions could be attributed to uncertainties in the constitutive parameters as well as manufacturing tolerances in the prototype fabrication. These slight mismatches result into mild reductions of the measured cloaking dip and bandwidth. This latter effect, attributable to unmodeled variations of the surface reactance away from the nominal design frequency, is more evident in the case of the dielectric cylinder, where the difference between measurements and simulations can reach ~5 dB outside the cloaking region. The disagreement might also be attributable to the poor electrical contact between the cylinder ends and the waveguide plates, which is expected to introduce parasitic effects and to modify the surface response. Nevertheless, our waveguide measurement setup proved to be capable to detect changes in the SW that can span a two-order-of-magnitude dynamic range.

Overall, it is remarkable that the mantle cloak, originally designed for plane-wave (far-field) illumination, performs well even in the near-field region, in spite of the possible setup errors of the in-house waveguide realization, the potential coupling effects due to the close proximity between the metasurface and the probe antenna, and to the unavoidable imperfections in the fabricated prototypes.

## Methods

### Prototype Fabrication

The dielectric prototypes (cf. Fig. 3a) are cylinders of radius 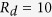 mm and height 10 mm, made of low-permittivity dielectric material. The mantle cloak was realized as a flexible metasurface made of three 17 *μ*m-thick copper strips on a 100 *μ*m-thick adhesive Kapton (GT7600) substrate.

The conducting prototypes (cf. Fig. 3b) are aluminum cylinders of radius 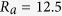 mm and height 10 mm. In this case, the mantle cloak is a multi-layer structure made of a 6 mm-thick layer of epoxy resin and a flexible metasurface (same materials and thicknesses as above) composed of 12 square patches.

### Mantle-Cloak Design and Numerical Simulations

The mantle-cloaks were designed following the procedure in ref. 65. First, we considered idealized structures (with the metals assumed as perfectly conducting and the metasurfaces modeled as homogeneous reactance sheets), and solved analytically the TEM plane-wave scattering problem by means of Fourier-Bessel-type expansions. In these models, we assumed 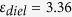 and 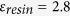 for the dielectric-core and resin relative permittivies, respectively, thereby neglecting the slight dispersion and losses. Moreover, we also neglected the Kapton layer in view of its small thickness. This allowed us to determine (via parameter scanning) the ideal value of the surface reactance 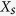 that minimize the SW. We then exploited the analytical formulas in ref. 65 to design strip-type and patch-type metasurfaces that would exhibit the required surface reactance value at the nominal design frequency 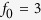 GHz. In this process, we determined the geometrical parameters *w* and *g* in Fig. 3 as well as the number of unit cells, also enforcing that an integer number of unit cells would exactly fit the exterior cylindrical surface of the scatterer. Finally, we fine-tuned these geometrical parameters via full-wave numerical simulations.

In particular, for the dielectric cylinder, the analytical model predicted an inductive surface reactance 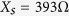, from which we derived 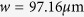, 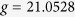 mm, and three unit cells. The final parameters in Fig. 3a caption derived from a subsequent fine-tuning based on full-wave numerical simulations by means of the commercial software COMSOL Multiphysics (www.comsol.com). In particular, we solved a 2-D plane-wave scattering problem by using the RF Module (MUMPS solver), with a maximum mesh size of 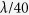 and perfectly-matched-layer terminations, resulting into over one million degrees of freedom. In these simulations, a finite conductivity 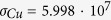 S/m was assumed for the copper strips and, once again, the thin Kapton layer was neglected.

For the conducting cylinder, a capacitive surface reactance 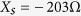 was predicted by the analytical model, which resulted into 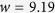 mm, 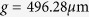, and 12 unit cells. This time, the fine-tuning implied solving a computationally more expensive 3-D problem (one unit-cell along the *z*-axis), and was carried out by means of the commercial software CST Microwave Studio (www.cst.com), yielding the final parameters in Fig. 3b caption. In particular, we utilized the time-domain solver, with a maximum mesh size of 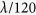, and with perfectly-matched-layer terminations in the 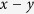 plane and periodic boundary conditions along the *z*-direction, resulting into over 25 million degrees of freedom. In these simulations, besides the already mentioned constitutive parameters and assumptions, 
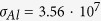
 S/m was assumed for the aluminum cylinders.

### Waveguide Measurements

The waveguide measurement setup, schematically illustrated in Fig. 1, is designed to operate within the 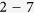 GHz frequency range. The waveguide is composed of two 1 m × 1 m aluminum sheets that are held parallel to each other via dielectric spacers around their perimeter. The height of the waveguide is 10 mm, which allows to support the dominant TEM mode only, as all other modes are evanescent at frequencies below 15 GHz. As previously mentioned, this effectively reproduces a 2-D scenario, as the electric and magnetic fields are mostly invariant along the *z*-axis. Therefore, a field map at any plane within the waveguide should provide an equivalent characterization of the in-plane scattering.

To better emulate the ideal conditions of an infinite waveguide in the 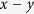 plane, its sides are surrounded and terminated with tapered foam microwave absorbers having a thickness comparable with the waveguide height. The absorbers are cut into a saw-toothed pattern in order to reduce unwanted reflections from the waveguide ends and in close proximity of the source. Small sags along the planar directions that may cause artifacts into the field maps reconstruction are minimized by bolting aluminum bars to the upper plate until the entire structure is flattened.

A small monopole antenna is placed at one end of the waveguide in a fixed position. This is a line-source-like excitation, yielding cylindrical wavefronts in the waveguide. To measure the electric field distribution inside the waveguide, a small semi-rigid coaxial cable is used, with its electrically thin center conductor exposed. This cable is placed in the vertical direction, with a length that almost matches the waveguide height, so as to maximize the collection of the electric field 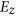. Since its transverse dimension is subwavelength, it has a minimal perturbation on the fields inside the waveguide.

The scatterer to be characterized is placed in the waveguide at a center-to-center distance 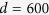 mm (i.e., between 4 and 14 wavelengths, over the operational frequency range) from the monopole source antenna. Since the transverse size *D* of the scanned area is much smaller than the source distance *d*, the cylindrical wavefront can be approximated by a planar one, with a phase difference 
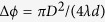
 always less than 10%, when it hits the scatterer and the surrounding region.

A translation stage moves the probe in the 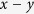 plane, allowing the scanning of a 70 mm × 50 mm area. The linear stages have a step resolution of 1 mm, resulting in field maps typically having 70 × 50 data points. The VNA (HP 8720C) provides the source microwave signal and phase sensitive detection of the return signal. A customized LabView (www.ni.com/labview) code drives the motion of the stages, and allows to record the data samples measured by the VNA. Each spatial step of the probe is followed by a hold state, during which the transmission-coefficient 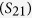 data are stored as complex values.

## Additional Information

**How to cite this article**: Vitiello, A. *et al.* Waveguide Characterization of S-Band Microwave Mantle Cloaks for Dielectric and Conducting Objects. *Sci. Rep.* **6**, 19716; doi: 10.1038/srep19716 (2016).
